# The Meaning of Living in the Time of COVID-19. A Large Sample Narrative Inquiry

**DOI:** 10.3389/fpsyg.2020.577077

**Published:** 2020-09-17

**Authors:** Claudia Venuleo, Tiziana Marinaci, Alessandro Gennaro, Arianna Palmieri

**Affiliations:** ^1^Department of History, Society and Human Studies, University of Salento, Lecce, Italy; ^2^Department of Dynamic and Clinical Psychology, Sapienza University of Rome, Rome, Italy; ^3^Department of Philosophy, Sociology, Education and Applied Psychology (FI.S.P.P.A.), University of Padua, Padua, Italy

**Keywords:** COVID-19 pandemic, Semiotic Cultural Psychosocial Theory (SCPT), sense-making, narratives, symbolic universes, cultural milieu, Italy

## Abstract

The spread of the COVID-19 pandemic has been a sudden, disruptive event that has strained international and local response capacity and distressed local populations. Different studies have focused on potential psychological distress resulting from the rupture of consolidated habits and routines related to the lockdown measures. Nevertheless, the subjective experience of individuals and the variations in the way of interpreting the lockdown measures remain substantially unexplored. Within the frame of Semiotic Cultural Psychosocial Theory, the study pursued two main goals: first, to explore the symbolic universes (SUs) through which Italian people represented the pandemic crisis and its meaning in their life; and second, to examine how the interpretation of the crisis varies over societal segments with different sociodemographic characteristics and specific life challenges. An online survey was available during the Italian lockdown. Respondents were asked to write a passage about the meaning of living in the time of COVID-19. A total of 1,393 questionnaires (mean = 35.47; standard deviation = 14.92; women: 64.8%; North Italy: 33%; Center Italy: 27%; South Italy: 40%) were collected. The Automated Method for Content Analysis procedure was applied to the collected texts to detect the factorial dimensions underpinning (dis)similarities in the respondents’ discourses. Such factors were interpreted as the markers of latent dimensions of meanings defining the SUs active in the sample. A set of χ^2^ analysis allowed exploring the association between SUs and respondents’ characteristics. Four SUs were identified, labeled “Reconsider social priorities,” “Reconsider personal priorities,” “Live with emergency,” and “Surviving a war,” characterized by the pertinentization of two extremely basic issues: what the pandemic consists of (health emergency versus turning point) and its extent and impact (daily life vs. world scenario). Significant associations were found between SUs and all the respondents’ characteristics considered (sex, age, job status, job situation during lockdown, and place of living). The findings will be discussed in light of the role of the media and institutional scenario and psychosocial conditions in mediating the representation of the pandemic and in favoring or constraining the availability of symbolic resources underpinning people’s capability to address the crisis.

## Introduction

The spread of the COronaVIrus Disease 2019 (COVID-19) has been a sudden, disruptive event that has strained the health system and had huge repercussions both on the social and economic plane and at the individual level. The containment of the massive outbreak of the virus strained international and local response capacity and distressing local populations. With no established treatment or vaccine to contain the infection rate among the population and not overload the often-limited health systems, most of the affected countries implemented emergency lockdown procedures through mass quarantine.

In Italy—the second country worldwide after China to be massively hit by the crisis (to date, as many as 238,159 reported cases and 34,514 deaths have resulted from COVID-19 in this country—Bulletin of the integrated supervision of the Istituto Superiore di Sanità, and Istituto Nazionale di Statistica, 2020, updated 19 June 2020)—lockdown measures were established by the Government to contain the infection rate and applied first to the so-called “red zone” (Lombardia and 14 provinces of Veneto, Emilia Romagna, Piemonte and Marche) and then to the whole country (Decree of the President of the Council of Ministers, 9 March 2020). As a result, social contacts, entrenched habits, and daily routines were interrupted as never before: people stopped visiting relatives and friends; praying in churches; doing sports in the gym and in parks; visiting museums; attending cinemas, theaters, bars, and restaurants; participating in social and cultural events; taking a walk; or shopping.

Different scholars emphasized the potential psychological distress produced in citizens by this sharp breakdown of their habits and routine (Liu et al., 2020; Sood, 2020; Suresh, 2020; Vijayaraghavan and Singhal, 2020). For instance, the study by Liu et al. (2020) among the Chinese population found that 44.6% of the people were anxious about the unknown situation and their health, 33.2% suffered from stress due to the biodisaster, and more than half exhibited mild depression, acute stress, and anxiety. A recent review on studies that analyzed the psychological impact of quarantine at the time of previous pandemics—severe acute respiratory syndrome (SARS), Ebola virus disease, Middle East respiratory syndrome, swine flu (H1N1), and equine flu (Brooks et al., 2020)—reports symptoms such as confusion, anger, sleeping problems, and even symptoms of posttraumatic disorder (anxiety, bad memories, irritability and depression) related to the isolation and the break in routine. High degrees of social insecurity, in addition to the health hazards (Pellecchia et al., 2015), tensions within households (Di Giovanni et al., 2004), stigma, and psychosomatic distress (Lee et al., 2005), were also reported with regard to previous epidemics.

On the other hand, the overriding focus on the negative effects of the health emergency, although crucial, presents two main limitations. First, it may not allow the researchers to understand what kind of symbolic resources (i.e., worldviews, beliefs, modes of feeling, thinking, and acting) citizens mobilized in response to the acute stage of the pandemic and whether these resources were suited to support the management of the crisis in its whole breadth and depth. Second, it provides limited insight into variations in the experience of quarantine due to individual factors and social situations; negative psychological outcomes could be strongly influenced by contextual aspects related to the microsphere, such as with whom one lives and the quality of the relationship, as well as the macro social sphere (e.g., degree of trust in politics and/or science or kind of media information). For instance, the findings of a study based on qualitative semistructured interviews with community informants and households during Ebola (Caleo et al., 2018) emphasizes the importance of the community having a role in tailoring outbreak responses to make norms more acceptable and effective, as well as in the clear communication of complex health messages. In short, researchers have taken for granted that the pandemic was a psychological tsunami for individuals and that the tsunami was intrinsically determined by the pandemic as disruptive events that can only produce a disruptive impact on daily life, people’s psychosocial conditions, and circumstances. On the other hand, negative or difficult life events may provide special opportunities for meaning making (e.g., King et al., 2000; McLean and Pratt, 2006; Bakker, 2018) and for turning crisis into opportunity.

Surprisingly, little research has been conducted to understand the everyday experience (feelings, experiences, practices, actions) and perspectives of those affected by the lockdown measures for the COVID-19 crisis, as well previous epidemic (Cava et al., 2005; Braunack-Mayer et al., 2013). To our knowledge, currently no studies have been performed in Italy, or worldwide.

According to the outline considerations, the present work, within the frame of Semiotic Cultural Psychosocial Theory (SCPT), aims to explore the way Italian people represented the pandemic crisis and its meaning in their life, within the general view that pandemics do not have an invariant psychological meaning, but the opposite: they are the meaning by which people interpret their being-in-the-world to explain their reaction to the crisis. A brief outline of the SCPT will be provided, in order to frame the following analysis of psychosocial processes underpinning people’s current response to the pandemic crisis.

## Theoretical Framework

The SCPT (Valsiner, 2007; Salvatore et al., 2009, 2019c,d; Salvatore and Venuleo, 2013, 2017; Salvatore, 2018; Russo et al., 2020; Venuleo et al., 2020a) postulates the mediational role of sense-making in the way people represent and face their material and social world and in so doing shape their experience. Accordingly, people do not represent and respond to the reality of the pandemic as if it were the same states of affairs for everyone. Rather, each person interprets the pandemic in terms of specific meanings that are consistent with the symbolic universe (SU) grounding their own self and their being-in-the-world (Salvatore et al., 2018; Venuleo et al., 2020b). SUs are conceptualized as systems of implicit, only partially conscious, embodied generalized assumptions or patterns of meanings (significance, texts, practices, behavioral scripts) that foster and constrain the way the sense-maker interprets any specific event, object, and condition of their life (Salvatore et al., 2018). An example is provided by the generalization of the friend–foe schema, which implies that the whole variability of the circumstances is reduced drastically to just the one degree-of-freedom distinction between being or not being other-than-us.

People vary in their tendency to make use of generalized meanings (Feldman, 1995; Barrett et al., 2001; Barrett, 2006). According to SCPT, the capacity of the SU to promote adaptive responses is a function of the variable degree of salience of the generalized meanings composing them (Venuleo et al., 2020b). Whereas a high salience of the generalized meanings corresponds to a rigid, polarized, way of thinking, producing homogenizing affect-laden interpretations of the reality (typically organized by the bad/good, pleasure/displeasure opposition), a low salience corresponds to more flexible thinking, able to capture the distinct events of the experience and to produce differentiated meanings that favor the process of learning from experience. A similar concept was expressed by Barrett et al. (2001) when they suggest that people vary in their capacity of emotional differentiation and argue that individuals with highly differentiated emotional experience are better able to reflectively regulate emotional experience to inform adaptive responses. With reference to the current pandemic crisis, different scholars have observed how fear and, more broadly, a general state of anxiety (e.g., of getting infected and/or of infecting someone else, of losing friends or relatives, of being alone, of not “making it” economically—the fear that nothing will ever be like before) was the dominant emotional reaction of the society to the pandemic crisis (Casale and Flett, 2020; Presti et al., 2020; Schimmenti et al., 2020). It is the common response to conditions and events that are a major violation of the expected state (e.g., Proulx and Inzlicht, 2012; for a review, see Townsend et al., 2013; for an analysis of the emotional response to a pandemic, see Kim and Niederdeppe, 2013) and can be interpreted as the marker of high affective activation: it produces global, homogenizing, and generalizing embodied affect-laden interpretations of reality, at the cost of more fine-grained and differentiated analytical thought (Venuleo et al., 2020a). Among other manifestations, these high affect-laden interpretations are expressed though the spreading of conspiracy theories (and the related devaluation of experts’ knowledge) and the initial blaming of specific outgroups (“the Chinese,” or the “immigrants,” in some populist propaganda), based on the friend–foe schema, which influenced alarmist comments and discourses on the social media (Venuleo et al., 2020a). Less polarized and more flexible interpretations may be indicated by discourses focused on the need to learn from the pandemic what can usefully be changed in past choices and habits to better manage personal and/or societal resources and construct a better future (for one’s own life and/or, more broadly, for the life of society), as well as in the initiatives activated to mobilize relational resources and create a dense solidarity network.

According to SCPT, the SUs through which people’s sense-making is expressed are not transcendental intrapsychic structures, but in their working depend on sociohistorical conditions and are placed within the sphere of social discourses, which suggest what a particular event consists of, why it became a disaster, who was responsible, what should be learned from it (Fairclough, 1992; Ratner, 2008; Venuleo and Marinaci, 2017; see also Cannon and Müller-Mahn, 2010). Broader contextual dimensions (e.g., ideologies; shifting frameworks of knowledge; power structures; health and economic policies; the discourse of the media, scientists, and politicians) such as psychosocial conditions impose constraints on the multiple ways people could make sense of the events, problems, and circumstances of their life (Salvatore and Zittoun, 2011; Venuleo and Marinaci, 2017; Marinaci et al., 2019).

Framing with SCPT, thus, the “pandemic” can be considered not only a sign referring to an actual event, but a *hyperdense* polysemic *sign* (Venuleo et al., 2020a). By *hyperdense*, we mean a sign that stands for the whole of social life, due to the first tenet deriving from SCPT cited above: each person interprets the actual event of the pandemic in terms of specific meanings that are consistent with the SU grounding his/her own self and his/her being-in-the-world. By polysemic, we mean a sign that can be interpreted in very different manners and used within a great many discourses and social practices, with different cultural and psychosocial contexts (cf. Venuleo et al., 2020a): this aspect reflects the second tenet of SCPT: SUs depend on sociohistorical conditions. One therefore finds “pandemic” associated with signs such as war, enemy, and conspiracy, consistent with a paranoid affective interpretation of the social landscape, which characterizes a vast segment of the population in the contemporary scenario (Salvatore et al., 2018), or also one finds “pandemic” associated with signs such as solidarity, hope, reborn, and consistent with an interpretation of the crisis as a chance to learn from the experience and to make new choices for a better future; and so forth.

Previous studies have shown the essential role of SUs in grounding, motivating, and channeling social and individual behavior (Venuleo, 2013; Venuleo et al., 2015, 2017; Marinaci et al., 2019; Salvatore et al., 2019d; Venezia et al., 2019). Different interpretations are not merely abstract judgments—they are a way of being channeled to act and react in a certain way.

Accordingly, research into the interpretative categories that underpinned people’s responses during the pandemic is crucial for public health officials and policy makers in comprehending what favored or hindered an adaptive response to the crisis, in order to outline exit strategies and to design more effective future health emergency plan.

## Aims of the Study and Hypotheses

On the basis of the theoretical premises discussed above, the study aims to explore the SUs through which people represented the pandemic crisis and its meaning in their life. The following hypotheses guided the study.

First, based on the theoretical frame of SCPT, stating the dependence of the SUs on the cultural and psychosocial contexts people belong to, we expect that a plurality of representations of the crisis scenario is active in the cultural milieu. Particularly, we expect that highly rigid/polarized and homogenizing affect-laden interpretations of the pandemic crisis framing it in terms of a battle against an uncertain and unknown enemy and the loss of a prior idealized state (e.g., loss of life, freedom, habits) emerge along with more flexible representations (e.g., pandemic as opportunity to change), reflecting people’s variability in the categorization of the experience (Barrett et al., 2001; Salvatore et al., 2018) and the variability of the media and social media discourses characterizing the cultural milieu.

Second, we expect SUs to vary over social segments, because of the variability of psychosocial conditions, discourses, and social practices, which people are exposed to during the pandemic. Specifically, we explore the role of respondents’ sociodemographic characteristics—such as sex, age, and job status—which we expect to be related to specific life challenges and health, social, and economic concerns—and social characteristics related to the health emergency, such as work situation during the pandemic and place of living (having different characteristics regarding the spread of the virus and health and media alarm).

## Materials and Methods

Narrative inquiry was chosen to gain access to the Italian people’s subjective experience of the health emergency. According to the definition of McAdams (2011), the story is a selective reconstruction of the autobiographical past and a narrative anticipation of the imagined future that serves to explain, for the self and others, how the person came to be and where his/her life may be going. Social researchers argue that personal narratives can capture particular attitudes, beliefs, and values about themselves as individuals (Baxen, 2008) and their ways of making sense of social experience and of their own role in it, as well as mirroring the changing social conditions (Bertaux, 1981) and elucidating processes of social change and the place of individuals within them (Andrews, 2007). In the terms of Gergen (1985), narratives are important because they are the means by which people understand and live their lives and because they are ways to participate actively in the practice of a particular culture.

The narratives used in this article were collected as part of the first phase of a mixed-methods research project aimed to analyzing the impact of the COVID-19 health emergency on everyday life. In the first phase, the subjective experience of people living in the time of COVID-19 was investigated, along with their social conditions and sociodemographic characteristics. In the second phase of the research (currently in progress), people were asked to keep a diary periodically to talk about the meaning of the pandemic scenario in their life.

### Instruments

An anonymous online survey was designed to assess feelings, emotions, and evaluation of the lockdown measures. The survey was available online from April 1 to May 19, 2020, coinciding with the government decree “Chiudi Italia” and disseminated through social networks.

People were asked to respond to the following question: *Imagine telling someone in the future who has not lived through this period what it meant for you to live in the time of COVID-19. What would you tell them?* They were encouraged to writing down everything that comes to mind with respect to the situation and responding in the manner that is deemed most appropriate, taking into account that the objective of the investigation was to collect people’s subjective experience.

Then, sociodemographic and social characteristics of respondents (i.e., sex, age, job status, job situation in the current pandemic scenario, and place of living) were collected.

All procedures performed in the study were in accordance with the ethical standards of the institutional research committee and with the 1964 Helsinki Declaration and its later amendments or comparable ethical standards. According to the ethical code of the Italian Psychology Association^1^ and the Italian Code concerning the protection of personal data (legislative decree no. 101/2018), participants were informed about the general aim of research, the anonymity of responses, and the voluntary nature of participation and signed an informed consent. No incentive was given. The project was approved by the Ethics Commission for Research in Psychology of the Department of History, Society and Human Studies of the University of Salento (protocol no. 53162 of April 30, 2020).

### Participants

A total number of 1,393 questionnaires and related texts (mean = 35.47, standard deviation = 14.92, women: 64.8%; North Italy: 33%, Central Italy: 27%, South Italy: 40%) were collected (Table 1).

**TABLE 1:** Sociodemographic characteristics of the respondents.

Variables		Frequency (%)
Sex	Men	491 (35.2%)
	Women	902 (64.8%
Age range (y)	18–25	582 (41.8%)
	26–35	269(19.3%)
	36–45	151(10.8%)
	46–55	192 (13.8%)
	56–65	152 (10.9%)
	>65	47 (3.4%)
Job status	Student	492 (35.3%)
	Employee	564 (40.5%)
	Self-employed	135 (9.7%)
	Precarious worker	34 (2.4%)
	Unemployed	112 (8.0%)
	Retired	56 (4.0%)
Job situation during lockdown	Ordinary	180 (12.9%)
	Working from home	354 (25.4%)
	Reduced hours	69 (5.0%)
	Suspended	175 (12.6%)
	Lost job	9 (0.6%)
	Not classified	606 (43.5%)
Residence	North	456 (32.7%)
	Center	380 (27.3%)
	South	557 (40.0%)

## Data Analysis

The analysis aimed to map the main *dimensions of meanings* underpinning the set of contents of the narratives collected and defining the SUs through which respondents make sense of their COVID-19 experiences. Each dimension of meaning can be conceived of as a generalized meaning component that was highlighted by the interviewees to talk about the time of COVID-19 and that provides space for a plurality of statements and positions. For instance, if the interviewees highlighted the challenges the pandemic brought to their life, then this dimension provides space to express different views/connotations on this aspect (e.g., some interviewees might talk about the change occurring in the relationship with their children; others might describe the changes occurring in their conjugal relationship). Thus, the meanings map goes beyond the descriptive level of content analysis and identifies the latent meanings generating the variability of the contents (for a similar approach, see Visetti and Cadiot, 2002; Venuleo et al., 2018a, b, 2019). To this end, an automatic procedure for content analysis [Automated Method for Content Analysis (ACASM); Salvatore et al., 2012; Salvatore et al., 2017], performed by T-LAB software (version T-Lab Plus 2020; Lancia, 2020), was applied to the whole corpus of texts obtained through the narratives. The method is grounded on the general assumption that the meanings are shaped in terms of lexical variability. Accordingly, a word such as “father” might, for instance, contribute to the construction of the symbolic meaning of “authority” if it is associated with other words such as “order,” “punishment,” “power.” Otherwise, the same word “father” might help to depict a different meaning, such as “protection” or “warmth,” if it is used together with other words such as “home” and “care.” A similar criterion of co-occurrence is entailed in the semantic differential technique (Osgood et al., 1957) and can be also equated to the free-association principle (Salvatore, 2014). Accordingly, the method of analysis applied to the textual corpus aims at detecting the ways the words combine with each other (that is, co-occur) within utterances, somewhat independently of the referentiality of the sentence (Lebart et al., 1998). ACASM procedure followed three steps.

First, the textual corpus of narratives was split into units of analysis, called elementary context units (ECUs). Second, the lexical forms present in the ECUs were identified and categorized according to the “lemma” they belong to. A lemma is the citation form (namely, the headword) used in a language dictionary, e.g., word forms such as “child” and “children” have “child” as their lemma. A digital matrix of the corpus was defined, having as rows the ECU, as columns the lemmas and in the cell *x*_*ij*_ the value “1” if the *j*th lemma was contained in the *i*th ECU; otherwise, the *x*_*ij*_ cell received the value “0,” Table 2 describes the characteristics of the dataset.

**TABLE 2:** Dataset.

	*N*
Texts in the corpus	1,393
Elementary contexts (EC)	3,531
Types	12,283
Lemma	1,122
Occurrences (Tokens)	139,883
Threshold of lemma selection	23
Lemmas in analysis	479

Second, a lexical correspondence analysis (LCA)—a factor analysis procedure for nominal data (Benzécri, 1973)—has been carried out on the obtained matrix, to retrieve the factors describing lemmas having higher degrees of association, that is, occurring together many times. Each factorial dimension describes the juxtaposition of two patterns of strongly associated (co-occurring) lemmas and, according to the model grounding the analysis (Salvatore et al., 2017; Gennaro et al., 2019, 2020), can be interpreted as a marker of a latent dimension of meanings underpinning dis(similarities) in the respondents’ discourses and defining their SUs. The interpretation of the factorial dimensions is carried out in terms of inferential reconstruction of the global meaning envisaged by the set of co-occurring lemmas associated with each polarity, based on the abductive logic of interpretation of the relationships among single contents/lemmas (Salvatore, 2014). The first two factors extracted from LCA were selected, as the ones explaining the broader part of the data matrix’s inertia, and labeled by three experienced researchers, in double-blind procedure, on the basis of the specific vocabulary and sentences composing the factors. Disagreement among researchers was overcome using a consensus procedure (Stiles, 1986).

The LCA provides a measure of the degree of association of any respondent with every factorial dimension, expressed in terms of respondent’s position (coordinate) on the factorial dimension. Accordingly, the SU the respondent belongs to is detected in terms of their factorial coordinates. In the final analysis, these coordinates reflect the respondent’s positioning with respect to the oppositional generalized meanings sustaining the SUs identified by the study. Once the coordinates of each subject were identified—as the third step—a set of χ^2^ analysis allowed us to explore the association between SUs and the respondents’ characteristics. For a more accurate reading, adjusted standard residuals were considered a *post hoc* procedure for statistically significant omnibus χ^2^ test (Agresti, 2007). Residuals represent the difference between the observed and expected values for a cell. The larger the residual, the greater the contribution of the cell to the magnitude of the resulting χ^2^ value obtained. Adjusted standard residuals are normally distributed; thus cells having absolute value greater than the critical value *N* (0,1), 1 - α/2 = 1.96 will have raw *p*-value less than 0.05 (for two-sided test). In so doing, *post hoc* hypotheses tests on standardized residuals were tested.

## Results

### Dimensions of Meanings and Descriptions of SUs

In Tables 3, 4, the two factorial dimensions obtained from the ACASM procedure, and for each of their polarities, the lemmas with the highest level of association (*V* test), are reported, as well as their interpretation in terms of dimensions of meaning. Henceforth, we adopt capitals letters for labeling the dimensions of meaning and italics for the interpretation of polarities.

**TABLE 3:** LCA output.

Representation of the Pandemic Crisis			
Health emergency		Turning point	
Test value*	Lemmas	Test value*	Lemmas
−18.225	To die	14.566	Experience
−16.860	Death	14.054	To live
−16.860	Dead	13.608	Meaning
−16.514	Mask	13.164	To mean
−15.514	To close	12.440	Time
−15.294	Closed	11.446	To rediscover
−14.863	Glove	11.162	Life
−13.273	Virus	9.954	To discover
−11.470	China	9.873	Discovery
−11.226	Supermarket	9.641	Future
−10.943	To wear	9.185	For me
−10.819	Italy	8.457	To appreciate
−10.518	Shopping	8.309	Freedom
−10.492	Home	8.208	To reflect
−10.477	To exit	7.949	Period
−10.094	Shop	7.381	Importance
−9.300	Queue	7.302	Uncertainty
−8.972	Subway	7.281	Important
−8.544	To arrive	7.196	Small
−8.535	Nurse	7.133	To learn
−8.207	TV news	7.095	Values
−8.203	News	6.947	Reflection
−8.186	Elder	6.927	To slow down
−8.184	School	6.852	Social
−7.890	To enter	6.706	Moment

Characterization of the first factorial dimension. *Highest levels of association standard scores (*V* test).

**TABLE 4:** LCA output.

Pandemic impact			
Daily life		World scenario	
Test value*	Lemmas	Test value*	Lemmas
−13.196	Friend	23.481	Enemy
−12.189	To close	17.437	War
−12.060	Closed	15.110	Impotence
−11.497	Lesson	14.661	To die
−11.357	To exit	13.513	Virus
−10.757	Day	12.840	Worldwide
−10.565	School	11.746	To fight
−9.920	Mask	10.464	Crisis
−9.558	Exam	9.852	Economic
−9.454	Online	9.656	Death
−9.407	Shop	9.656	Dead
−8.985	Video call	7.941	Uncertainty
−8.935	Shopping	7.937	Our
−8.701	Boy	7.931	Pandemic
−8.617	Glove	7.752	To hit
−8.580	From home	7.730	Policy
−8.136	To pass	7.728	Unknown
−8.075	Undergraduate	7.448	Healthcare
−7.720	University	7.323	Country
−7.719	To study	7.243	Victim
−7.717	Time	7.198	Future
−7.678	Week	7.179	Elder
−7.567	Book	7.140	Economy
−7.556	To work	6.959	Front
−7.447	Morning	6.579	Fear

Characterization of the second factorial dimension. *Highest levels of association standard scores (V test).

FIRST DIMENSION. REPRESENTATION OF THE PANDEMIC CRISIS: *Health emergency* versus *turning point.* This dimension opposes two patterns of words that we interpret as the markers of two ways of representing the COVID-19 crisis (Table 3).

(−) *Health emergency*. On this polarity, lemmas focusing on a contagiousness (*virus*) that cross the nations (*China*, *Italy*, *to arrive*) and having a dramatic impact on health (*to die*, *death*, *dead*) co-occur with lemmas related to the changes imposed to contain the health emergency: changes in daily habits (*to wear*, *mask*, *glove*, *supermarket*, *queues*) and throughout contexts and domains of life (*to close*, *closed*, *home*, *school*, *shopping*, *shop*, *subway*).

(+) *Turning point*. On this polarity, the reference to uncertainty—which suggests a crisis of meaning, the feeling of not having categories to interpret what happens or what to do to cope with the *moment*—co-occurs with lemmas that suggest the idea of a process of discovering new meanings to life (*to live*, *meaning*, *to mean*, *to discover*, *to rediscover*, *discovery*, *to appreciate*, *to reflect*), which invest the individual domain (*lived*, *for me*) and social life (*social*), and allows one to review one’s priorities and values (*importance*, *important*, *time*, *life*, *future*, *freedom*, *values*).

SECOND DIMENSION. PANDEMIC IMPACT: *Daily life* versus *world scenario.* The second factor extracted opposes two patterns of lemma that we interpret as the marker of two different interpretative “lens” to evaluate the impact of the pandemic crisis (Table 4).

(−) *Daily life*. In this polarity, the lemmas seem to refer to the change occurring in daily life habits (e.g., the adoption of protection: *mask*, *glove*) and domains of experience such as education, working, and interpersonal relationships (*school*, *university*, *lesson*, *exam*, *to study*, *to work*, *friend*, *shop*, *online*, *video call*) due the lockdown measures (*to close*, *closed*). Temporal trackers (*morning*, *day*, *week*, *time*) evoke the idea of a change unfolding in a limited temporal horizon.

(+) *World scenario*. In this polarity, a world war scenario is evoked (*enemy*, *front*, *war*, *to fight*, *to hit*, *to die*, *death*, *dead*, *victim*), without spatial and temporal borders (*virus*, *pandemic*, *worldwide*, *future*), disrupting social life at different levels (*crisis*, *policy*, *healthcare*, *economy*). A feeling of fear and a sense of helplessness (*impotence*) is associated with this scenario which appears to escape from the very possibility of being represented (*unknown*).

### Symbolic Area

**FIGURE 1:**
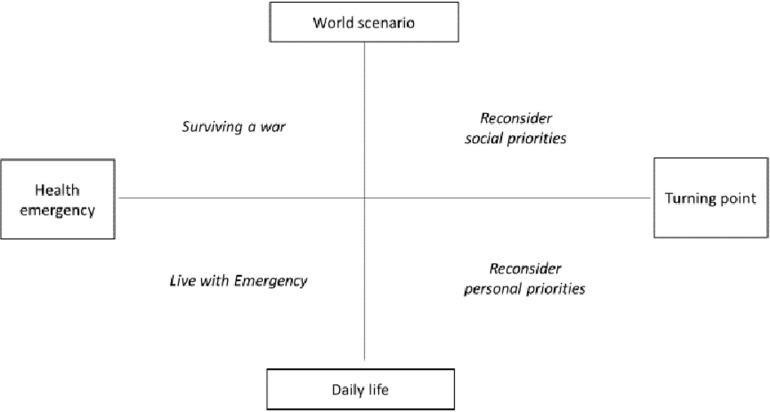
The symbolic space defined by the factorial dimensions.

The intersection between the two factorial dimensions identifies four quadrants, which we interpret in terms of SUs (henceforth SUs) (cf. Figure 1) and that were labeled: *Reconsider social priorities*, *Reconsider personal priorities, Living with emergency*, and *Surviving a war*. A description of each SU is reported below.

SU 1: *Reconsider social priorities*. This symbolic area is organized by a symbolization of the pandemic crisis as a “turning point” (right polarity of the first dimension) having an impact on the world scenario (upper polarity of the second factorial dimension). The pandemic here is recounted as something that transcends the health emergency and stands for something else—the by-product of a predatory and short-sighted way of conceiving human and social development, soliciting a reorganization of social values and priorities to build a better tomorrow. As such, the pandemic is shaped as a potential generative social turning point that can undermine the idea of invincibility of human beings, cast shadows on an idea of growth and progress measured in terms of technological and economic development, show the short-sightedness of our own policies, bring to light the connectivity among individuals and the being part of a collective, and help rediscover the importance of cooperation and solidarity. Examples of discourses are as follows:

Just a couple of months ago, we lived in an era where, as privileged spectators, we believed we were strong and invincible. Sitting in comfortable armchairs; many looked at the continuous natural disasters that occurred on the planet, with the strong and solid conviction that they would never touch our lives (…). One cold winter day, we woke up and without proper preparation, they told us that a virus was going to erase our hopes for tomorrow. Scientists, experts told us that we were wrong, that we were no longer the strongest (…). The virus had isolated us from the world, from our loved ones, had pushed us all together on a dangerous barge in a stormy sea, the same that for years had carried many migrants, alone, desperate, helpless, and needy (…). Everything has become fragile, in a few hours, the priorities have changed (…). If our boat is spared this stormy sea and we can survive this difficult test, we hope never to forget all this.

It led us to understand and reflect on the fact that we are not masters of the world! We always thought we were invincible, with our world made mostly of money, cutting-edge technology and comfort. But it is not true. Have we always had everything under control? No, never! When COVID-19 appeared, we may have begun to understand some of the non-material values that are the most important in addressing a pandemic of this kind properly and especially to consider our race, worthy of being called human! (…). In my opinion, the watchwords are solidarity, respect, understanding, listening, altruism, knowledge, and above all love.

I would talk about how the planet slowly began to breathe again (thanks to the closure of a lot of factories or various companies, or with the decrease in traffic). I would like to talk about how many people have rediscovered the Earth, the sacrifices, the fatigue, the fruits, and the satisfactions linked to it, thanks to working in the fields.

SU 2: *Reconsider personal priorities*. This symbolic area shares with the previous area a symbolization of the pandemic crisis as “turning point” (right polarity of the first dimension) but differs in the focus on the “daily life” impact (bottom polarity of the second factorial dimension). The pandemic is here recounted as sudden interruption of the ordinary, which leads to not taking for granted different aspects of life and being able to change significantly one’s perspective toward oneself and others, one’s way of being-in-the-world. The lockdown measures are experienced here and represented in their aspect of being a space–time suspension of routine, able to generate new meaning for experience and to reconsider values and priorities in life. Examples of discourses are as follows:

The being able to reclaim your time and your spaces.

Everything that used to be part of the normal routine becomes something out of the ordinary and no longer possible, and you are confronted, in an extremely profound way, with yourself.

My life was almost a boring routine, almost following a written script. COVID-19 forced me to reorganize my mental and physical spaces.

I would tell you about an experience of elasticity and resilience where the difference emerged starkly between those who had begun to work on themselves and those who, panicked, railed against the restrictions shifting the focus of their own problems (…). I would recount the rediscovery of some family tensions and wounds and the strengthening of the bond and love with my husband. (…) I would tell him that life always (sooner or later) presents us with challenges and that we must learn from them in order to grow and be better.

SU 3: *Live with emergency*. It is a symbolic area organized by a symbolization of the pandemic crisis as “health emergency” (left polarity of the first dimension) having an impact on “daily life” (bottom polarity of the second factorial dimension). Here the pandemic crisis, identified with a health emergency, is narrated by referring to the impact of the lockdown measures on personal everyday life, at different levels: change in daily habits to contain the risk of infection (e.g., wearing mask and gloves), management of overlapping roles at home due to the reorganization of school and work from home restriction on freedom of movement, and related feeling of fear and anxiety. The narration of what the pandemic has interrupted or has no longer made possible (e.g., “you can’t see”; “you can’t do”) is in the foreground. The pandemic is mainly seen in terms of loss of the previous condition/sphere of experience, which means that the interpretation of the new reality emerging from the pandemic rupture tends to be made within the affective grounds provided by the prerupture semiotic scenario. Examples of discourses are as follows:

A time where our certainty and habits changed, and the freedom of moving, traveling, and interacting with other persons was greatly limited. A time where the fear of getting sick made you suspect your neighbor and this inevitably changed everyday life, isolating and separating families and friends.

A bad time when you never feel safe when you leave the house and you always need to wear a mask and gloves: You can’t see your friends, you can’t do those normal things like having coffee in a bar, having dinner in a restaurant or having an aperitif. It’s spring, but we’re not enjoying it; we wanted to travel, see new cities or just be around the streets of our town, but you can’t do any of this.

At the beginning, the quarantine has me a bit destabilized; it meant giving up my everyday habits and my freedom of movement, but then I got strong, knowing that it was the only way to stop infection.

Period of anxiety, fear, and confinement. Privation of our freedom to safeguard people.

I have had to be the teacher and mother for my children aged 4 and 6 who have continued to follow the activities with online teaching (…) I don’t understand when I’m a mother or a teacher. My children have suffered so much being away from school and also the motivation to complete a task has fallen day after day. The work of encouragement and support was hard.

SU 4: *Survive a war*. It is a symbolic area organized by a symbolization of the pandemic crisis as “health emergency” (left polarity of the first dimension) having an impact on the world scenario (upper polarity of the second factorial dimension): a militaristic language is used to talk about COVID-19 and its impact on individual feeling and responses. *Tragic*, *terrifying*, and *frightening* are among the most connotations associated with a pandemic, lived as if it were an unexpected and unannounced war. The unpredictable character attributed to the crisis and its identification with an invisible virus whose space–time location, as well as physical drivers, is very hard to identify are associated with the feeling of being unprepared and helpless. Not being infected and surviving appear to be the only possible goals. Examples of discourses are as follows:

COVID-19 was a terrifying and unimaginable experience, maybe worse than a war because we fought with an invisible enemy, a virus, which has separated us from our loved ones for so long (…) a tragic and traumatic event for every country in the world, with many victims and as many healed.

Living in a pandemic is like living in a war, always with the uncertainty of being able to be saved, always with the fear for oneself and for others.

This is a tragic time that I had not budgeted for other than as one of the worst nightmares. The danger has come from far away, from China, in a subtle way, on the sly, and found us unprepared. First problem: how not to be infected? But many did not have time to ask themselves. I still have in my eyes the images of those in the ICU who died in complete solitude, the columns of army vehicles carrying the coffins, the churches full of coffins.

A nightmare.

### Relationships Between SUs and Respondents’ Characteristics

Table 5 reports the results of the χ^2^ tests applied to investigate the associations between SUs and respondents’ characteristics. Significant differences were found in all the characteristics.

**TABLE 5:** Association between symbolic universes and respondents’ characteristics.

Symbolic universes								
	Respondents’ characteristics		Reconsider social priorities	Reconsider personal priorities	Live with emergency	Surviving a war	χ^2^**	p***
	Men	n	178	*99*	*75*	*115*	*12.168*	*0.007*
Sex		Adj. res.	2.4	1.5	−1.2	−2.8		
	Women	n	279	158	165	281		
		Adj. res.	−2.4	−1.5	1.2	2.8		
Age range (y)	18–25	n	172	91	105	199	*41.466*	*0.000*
		Adj. res.	−2.3	−2.4	0.6	4		
	26–35	n	103	36	48	75		
		Adj. res.	2.1	−2.4	0.3	−0.3		
	36–45	n	54	32	24	36		
		Adj. res.	0.8	0.9	−0.4	−1.3		
	46–55	n	66	47	39	35		
		Adj. res.	0.4	2.3	1.2	−3.4		
	56–65	n	47	39	19	40		
		Adj. res.	−0.4	2.6	−1.6	−0.5		
	> 65	n	15	12	5	11		
		Adj. res.	0.1	1.5	−1.1	−0.5		
Job status	Student	n	143	80	84	172	*26.628*	*0.018*
		Adj. res.	−2.3	−1.6	−0.2	3.9		
	Employee	n	195	103	111	139		
		Adj. res.	1.1	−0.2	2	−2.6		
	Self-employed	n	46	29	17	37		
		Adj. res.	0.5	1	−1.4	−0.2		
	Precarious worker	n	14	7	3	9		
		Adj. res.	1.1	0.3	−1.3	−0.3		
	Unemployed	n	44	22	18	25		
		Adj. res.	1.5	0.3	−0.4	−1.5		
	Retired	n	15	16	7	14		
		Adj. res.	−0.8	2.2	−0.8	−0.4		
Job situation during	Ordinary	n	63	48	26	36	*27.928*	*0.22*
the lockdown		Adj. res.	0.8	3.1	−1	−2.6		
	Working from home	n	121	57	67	98		
		Adj. res.	0.6	−1.3	1	−0.4		
	Reduced hours	n	28	15	12	13		
		Adj. res.	1.3	0.7	0	−1.9		
	Suspended	n	59	30	34	46		
		Adj. res.	0.3	−0.5	0.9	−0.6		
	Lost job	n	4	2	0	2		
		Adj. Res	1	0.4	−1.3	−0.3		
	Not classified	n	182	105	101	201		
		Adj. Res	−2	−1	−0.5	3.4		
Living place	North	n	153	63	74	151	*19.104*	*0.004*
		Adj. res.	0.5	−3.1	−0.7	2.8		
	Center	n	117	71	77	108		
		Adj. res.	−1.2	0	1.7	−0.2		
	South	n	187	123	89	137		
		Adj. res.	0.7	3	−0.9	−2.5		

*Adj., adjusted standard residuals; ^∗∗^chi-square; ^***^p-value.

Concerning gender (χ^2^ = 12.168, *df* = 2, *p* < 0.05), the adjusted standardized residuals show that men were more associated with “Reconsider social priorities” SUs, whereas women were more represented in “Surviving a war.” Concerning age (χ^2^ = 41.466, *df* = 15, *p* < 0.000), respondents aged 18–25 years mostly represented the COVID-19 experience as surviving a war, respondents aged 26–35 years experienced COVID-19 as an opportunity to reconsider social priorities, and respondents 46–55 and 56–65 years assumed the lockdown in terms of reconsidering personal priorities.

With respect to job status (χ^2^ = 28.628, *df* = 15, *p* < 0.05), retired persons tend to represent the crisis scenario as a turning point, leading to reconsider personal priorities, employees in terms of living with the emergency, and students in terms of surviving a war. With respect to working during the health emergency (χ^2^ = 27.928, *df* = 15, *p* < 0.05), individuals maintaining their ordinary work situation during the pandemic tend to experience the crisis scenario as an opportunity to reconsider personal priorities.

The three macro areas of Italy in which respondents live—northern Italy, central Italy, and southern Italy—showed significant difference (χ^2^ = 19.104, *df* = 6, *p* < 0.05) in the opposition among northern part versus southern part: the former is more associated with surviving a war experience and the latter to reconsidering personal priorities.

In short, the highlighted differences allow us to obtain a clear picture of the respondents belonging to the different SUs retrieved: the representation of the COVID-19 crisis in terms of reconsideration of social priorities (SU 1) is represented by male respondents, aged 26–35 years, and the retired; “Reconsider personal priorities” (SU 2) characterizes people aged 46–55 and 56–65 years, retired, and maintaining ordinary work conditions and people of Southern Italy. The representation of the COVID-19 crisis in terms of *Living with emergency* (SU 3) characterizes employees, whereas *Surviving a war* (SU 4) characterizes women, people aged 18–25 years, students, and people living in the north of Italy.

## Discussion

The first goal of the study was to explore the SUs through which Italian people represented the pandemic crisis and its meaning in their life. The analysis of the narratives based on the ACASM procedure led to the identification of four distinct SUs organized by two main dimensions of meaning, which foreground two very basic issues: what the pandemic crisis consists of (health emergency vs. turning point) and its extent and impact (daily life vs. world scenario).

Consistently with the hypothesis, more rigid/polarized and highly homogenizing affect-laden interpretations, triggering feelings of fear and anxiety and framing the pandemic crisis as a battle against an uncertain and unknown enemy and/or the loss of a prior idealized scenario (SUs labeled “Surviving a war” and “Living with an emergency”), emerged along more flexible representations (SUs labeled “Reconsider social priorities” and “Reconsider personal priorities”), reflecting the variability of the media and social media discourses, which seem to characterize the cultural milieu.

Specifically, the SUs labeled “Surviving a war” and “Living with an emergency” differ with regard to the identification of the pandemic crisis as a social or individual rupture but share a short-term representation of the changes imposed by the pandemic related to a focus on the health emergency (more than a crisis encompassing health, economic, political, and social levels of analysis), which brings to the foreground the dichotomy between life and death and between the “normal things” that the pandemic emergency has interrupted to safeguard people (“You can’t see your friends, you can’t have coffee in a bar, you cannot travel …”) and the extraordinary habits imposed by the crisis. The pandemic is thus identified as a sectorial and confined event, although frightening, which can almost trigger at the individual level a reorganization of one’s habits and routines to defend oneself and one’s loved ones, and at the societal level strong measures of restriction of people’s freedom to move to avoid overloading the health system. However, the pandemic does not seem to work as something new that calls for an accommodation of one’s way of interpreting one’s own life and the world scenario; rather, it is approached through categories that foreground the loss or the lack of what existed before the rupture. This kind of position lends itself to be interpreted as the marker of an intense affective activation that triggers a homogenizing form of thinking which represents the new according to the past (Bria, 1999; Salvatore and Freda, 2011; Salvatore and Venuleo, 2017). Indeed, to express concerns about what was missed or interrupted by the pandemic entails the instantiation/reiteration of the presence of what was before (the past scenario) as the canonical order according to which the present is interpreted. In the final analysis, the concern is an (unintentional) way of keeping a certain version of the self/world psychologically alive regardless of the changes occurring in the real world.

On the other hand, the view of the pandemic as a turning point—which characterizes the SUs labeled “Reconsider social priorities” and “Reconsider personal priorities”—identifies a different area of meaning, where the rupture opens to a new way of being-in-the-world, and is felt as an opportunity to reflect on previous choices and their critical impact and to make the future better. To use an image, people’s meaning-making seems to move from the focus on loss (e.g., the dead people that will never come back, or the daily habits interrupted)—which characterizes the previously discussed SUs—toward a gaze to the future, the new adjustment challenge that one has to address. What one can learn from the crisis and what has to be changed are represented differently. Whereas the turning point concerns the individual life (“Reconsidering personal priorities”), the pandemic as a rupture highlighted the fragility of life and led to the search for a new way of managing one’s time and a clearer consideration of what matters. Whereas the turning point concerns the social and public sphere (“Reconsidering social priorities”), the pandemic rupture highlighted the critical impact of short-term and local politics and the need for more awareness of the interdependence among people and countries, which could facilitate reorganization of previously considered out-groups and in-groups into a single community with a common destiny.

As to Hypothesis 2—the interpretation of the crisis varies over societal segments with different psychosocial characteristics—the findings showed that significant associations exist between SUs and all the respondents’ characteristics considered (sex, age range, job status, job situation during lockdown, and place of living).

It is worth noticing the differentiated position of women, young adults (aged 18–25 years) and students compared respectively to men, adults aged 26–35 and 46–55 years, people maintaining their ordinary work situation during lockdown, or to the retired. The former tend to interpret the pandemic crisis as a health emergency, confronting people with the shared goal to survive, whereas the latter in terms of a personal or social turning point. Findings suggest that having a more stable life situation and less economic and job concerns could favor a more reflexive stance on the pandemic crisis. By contrast, unique challenges imposed by the lockdown measures, such as those related to the disrupted social roles and returning to living with parents, which may impact mainly students and emerging adults (Gruber et al., 2020), could have favored a interpretation of the crisis in terms of loss and urgency to return to the prerupture scenario.

As concerns the association between the SUs “Live with the emergency,” focusing on employees and the disruptive changes occurring in their personal daily life due to the lockdown measures, it can be interpreted considering how they were asked to close their offices and work from home (about 81% of the worldwide workforce has been affected by full or partial workplace closures, see Saviæ, 2020). Findings from recent studies show that working from home relates to the feeling of work intruding into personal life and work-life conflict (Molino et al., 2020), which could have triggered the daily stress and the feeling of living with and within an emergency.

The contrasting position of women and men deserves a comment, too. The negative impact of the coronavirus pandemic outbreak on equality (Bernardi, 2020), and particularly on gender equality, is recognized, although few detailed data are currently available (Kristal and Yaish, 2020). Data from the World Economic Forum (Hutt, 2020) show that women are responsible for the so-called unpaid care work three times more than men; it is likely that the care of children, the elderly, and other vulnerable groups was mostly provided by women also during the lockdown. With respect to Italy, the context of the current study, women tend to be the ones mainly responsible for the care of children in the family context. During the lockdown and the related closure of schools, and given also the insufficiency of the resources allocated to family support for children’s care, they have had to do a lot of multitasking and—often in the same space (the home)—to perform work assignments and activities related to the family management and teach their children (Rinaldi, 2020). This complex of circumstances may have triggered greater stress and more in general an affective activation of anxiety, foregrounding the risk of “losing the battle” (health, economics, social resources) more than the hope for a different future. Different exposure to health and media alarms may explain the differences related to the area of residence: people from North Italy tend to interpret the COVID-19 crisis as a war to which one has to survive, whereas people from South Italy as a personal turning point. It is not surprising. The expansion of the COVID-19 outbreak began in northern Italy, where the higher incidence of the coronavirus contagion is currently active and where the percentage of people infected and dead was far higher than in the rest of Italy (Santacroce et al., 2020). The daily bulletin of the data provided by the civil protection about the infected people and deaths and the media discourses depicting the overload of hospitals and of frontline health workers have contributed to depict a war scenario and to fuel feelings of fear and impotence. Fresh in everyone’s minds are the dreadful images—shown worldwide by the media—of the long rows of military trucks transporting the dead from the hospital outside the Lombard city of Bergamo (North Italy), because of lack of space to bury them in the town cemeteries.

Beyond the specificities of the associations detected between respondents’ characteristics and SUs, this kind of results shows how the meaning of the pandemic, the possibility that the crisis seems to be the loss of a previous desirable state of “normality” or a chance to rethink what went before and to generate new opportunities, is not ubiquitous and invariant but mediated by people’s sense-making.

On the other hand, as previously observed, according to SCPT, people’s affective interpretation of the pandemic scenario is not formed in a social vacuum. With regard to the interpretation of the pandemic scenario in terms of a mere health emergency and war against an unknown enemy, which forces government and individuals to fight for people’s survival (see SUs labeled “Surviving a war” and “Living with an emergency”), one can see its full continuity with the media and institutional discourses. Here the pandemic crisis was identified substantially with a health emergency and framed by affect-laden metaphors, with a clear prevalence of militaristic language: COVID-19 was widely depicted as an “enemy to defeat,” hospitals as “the trenches,” doctors and nurses as “heroes on the frontline,” and the counter-action against the virus as a “war” (Cassandro, 2020), as often found in the political and media discourses about previous epidemics (e.g., AIDS: Connelly and Macleod, 2003; SARS: Meng and Berger, 2008; Ebola: Trèková, 2015). Seminal studies argued that the use of militaristic language and metaphors makes it easier to sacrifice people and their rights (Fornari, 1970; Ross, 1986) and exculpate governments from responsibility (Larson et al., 2005), such as the kind of economic investment made in the health system and research. The Semiotic Cultural Psychology Theory suggests that affect−laden, simplified interpretations of the reality—such as those that underlie processes of enemization—restore the capacity of making sense of an uncertain social landscape (Venuleo et al., 2020a). From this standpoint, the fact that a high affect-laden interpretation of the pandemic scenario emerges in our analysis of how people make sense of this time of crisis is not surprising. The more the uncertainty of the scenario, the more sense-makers are likely to restore the stability of their sense-making through their adherence to generalized worldviews (Russo et al., 2020). Findings of studies based on the Terror Management Theory (Greenberg et al., 1997; Greenberg and Arndt, 2012) provide empirical support to this thesis. Recent studies among European societies reveal that about 40% of the respondents view the external world as if it were full of threats that may disrupt their living space (Salvatore et al., 2018). From this standpoint, the identification of the pandemic crisis as war appears to be only a further form reflecting the semiotic mechanism through which a lot of problems, critical changes, and ruptures (e.g., unemployment, worsening of living conditions, …) are currently mentalized by a large segment of the population in the current cultural milieu.

Unfortunately, we have not collected measures (e.g., people’s attitudes and compliance with the health measures) that allow us to empirically evaluate the impact of the different symbolic positions detected on the pandemic crisis; however, few speculative hypotheses can be made on the bases of previous studies. Scholars have suggested that when people are gripped by strong fear and feel that their survival is at stake, they are more likely to break their entrenched habits (Barrett et al., 2001; Coombs et al., 2007), a vital factor in coping with the emergency, as already found among other populations during previous pandemic such as the SARS (Hsu et al., 2006) and H1N1 pandemics (McVernon et al., 2011). With respect to the COVID-19 emergency, it is reasonable to think that the widespread fear of being “hit” (getting infected and/or of infecting someone else), of losing friends or relatives in the battle, favors higher levels of compliance among the Italian population than one might have expected if one considers the quite low level of trust in the institutions and commitment to the common good characterizing Italian communities (e.g., Salvatore et al., 2019a; Venuleo et al., 2020a). However, in the medium and long term, the fear response could increasingly prove to be inadequate in managing the pandemic: this is because the fear response persists insofar as the alarm trigger is active while prone to fade away as a result of desensitization. Thus, a global reduction of compliance with measures to contain infection can be expected to be associated with the flattening of the infection curve and of the decrease in the alarms launched by TV, newspapers, social media, and political speeches. Further studies are needed to examine this hypothesis in greater depth.

A further critical aspect of a symbolization of pandemic as a war against a virus is that it looks at the pandemic crisis as a sectorial and confined event, which can trigger short-term changes at the individual level (e.g., avoidance of social aggregations) and societal level (e.g., a greater investment in the health field), but not favor the holistic view required to empower individuals and institutional effort to learn from the crisis how to build a better tomorrow.

On the other hand, the view of the pandemic as a turning point—which characterizes the SUs labeled “Reconsider social priorities” and “Reconsider personal priorities”—identifies a different area of meaning, turning crisis into opportunity, involving a promise of some kind of progress toward better living conditions, opening one’s gaze to the future and leading people to search for a new way of managing their personal and societal resources. Specifically, conceived as a social turning point, the pandemic reveals the presence in the cultural milieu of a set of symbolic resources (e.g., meanings, cognitive schemas, values, social representations, attitudes, behavioral scripts, etc.) that foster the individual’s capacity to interiorize the collective dimension of life, what has been called *semiotic capital* (Salvatore et al., 2018; Venuleo et al., 2020a). Recent studies on the SUs active among European societies (Salvatore et al., 2018, 2019b) reveal that, along with a view of the external world as full of threats that can disrupt their living space, there are also SUs, although a minority in the cultural milieus, which recognize the systemic level of social life and the collective interest as something that matters, therefore the common good as a super-ordered framework of sense orienting individual decisions and actions. It is argued that semiotic capital is particularly important in the management of the pandemic scenario, because people will not only have to accomplish the task of complying with negative regulations (e.g., avoid social gatherings, keep a distance from other people), but—more profoundly, to integrate a reference to an *abstract common good*—the management of the risk of resurgence of the pandemic—in their mindsets, as a salient regulator of their way of feeling, thinking, and acting (Venuleo et al., 2020a). And this task requires people to be enabled to recognize and give relevance to the relation between the individual sphere of experience and the sphere of collective life and, as such, to go beyond the mere focus on the individual experience and interest (see also: Schimmenti et al., 2020).

### Implications for Policy

Typically, the focus on the psychological impact of the pandemic and related lockdown measures was accompanied by the emphasis on individuals’ need for psychologist and psychological support; suggested actions include support lines for anxious people, telecounseling, virtual connecting, and help groups (Sood, 2020). However, this approach, although crucial, does not appear to be enough to sustain the development within the population of the symbolic resources underpinning people’s capability to address the crisis. The pandemic demands that both the individual and society as a whole consider the consequences of particular choices and actions, a strategic issue that has implications far beyond the sphere of individual well-being and beyond the challenge of surviving the health emergency (which is in the foreground in SUs1).

We have above suggested that the impact of the pandemic crisis on individuals and their ability to respond adaptively to it are shaped by the social and cultural resources that they have to hand. This also means recognizing that disruptive events, like a pandemic, constitute not only natural hazards, but also socially constructed events: the product of the impact of a disruptive event on people whose vulnerability is also constructed by social, economic, and political conditions (see Cannon and Müller-Mahn, 2010). Counterfactual thinking in support to this thesis is that problems exponentially more disruptive than SARS-CoV-2, such as climate change at the societal level, or smoking at the individual level, have been unable to produce a reaction of fear even remotely like that of the pandemic. By extension, this means that the feeling of fear and impotence that have characterized a large part of the population are not a direct reaction to the pandemic as such, but to the way the crisis scenario has been perceived, discussed, and negotiated in the society. Obviously, this does not mean to question the seriousness of the pandemic emergency; rather, this perspective emphasizes how political decision making and discourses in the public sphere affect the way people make sense of what is happening and their feeling of being passive spectators or victims of an event beyond their control or also active agents and drivers of change.

Cultural manifestations can be addressed and, eventually, counteracted only if the cultural dynamics underpinning them are explained in their specific and contingent way of functioning (Russo et al., 2020). The characteristics of sense-making outlined by SCPT offer a contribution in that direction. More specifically, the fact that sense-making is embedded in affect−laden, generalized, holistic meanings (SUs) and in the cultural milieu and the performative quality of the processes can be translated into methodological criteria for designing strategies to support the cultural possibility of turning the pandemic crisis into a cultural opportunity. Although a deeper, systematic discussion of the methodological criteria that can be drawn from the theoretical framework is beyond the scope of this work, three speculative hypotheses can be considered, showing the heuristic and pragmatic potentiality of SCPT.

First, the acknowledgment of the holistic nature of the generalized meaning underpinning SUs implies that any intervention that restricts its action to the specific domain of health (in terms of fighting the virus) is likely to have limited efficacy, given that people shape their way of addressing the pandemic crisis and relate to sanitary measures not only according to health domain−specific beliefs, but also according to their global worldview that concerns the world of experience as a whole (Salvatore et al., 2019d).

Second, if the SUs develop within specific sociohistorical conditions and come alive in the context of discourse and interaction (Linell, 2009), we must also recognize the role of the way the crisis is managed at an institutional level and signified by communicative practices and discourses, which therefore have to be critically examined.

Third, the acknowledgment of the performative nature of sense-making leads us to recognize that SUs are not produced by statements but enacted by social practices and rooted in the social group’s mindscape. This entails that, to act on the cultural dynamics, policy does not have to espouse contents (beliefs, values, principles), but to design social practices that encapsulate those contents (Venuleo et al., 2020a). For instance, to promote the value of cooperation and solidarity, rather than advocating it, social practices grounded on the representation of otherness as a resource have to be implemented within the social group. First comes action; then meaning follows. More specifically, the promotion of semiotic capital is carried out through the design and activation of settings of social practices that encapsulate the worldviews, the beliefs, and the views of otherness making up the semiotic capital.

### Limitations and Future Direction of Research

The results of the present study should be considered in light of several methodological limitations. First, our case study is based on an Italian convenience sample; thus, the results cannot be generalized and have to be related to the specific cultural context under analysis. Because SUs depend on their working on sociohistorical conditions and are placed within the sphere of social discourses, we might suppose that, in other countries, other SUs emerge to represent the pandemic crisis and its impact.

Second, the analysis of how SUs vary over social segments due to the variability of psychosocial conditions could be improved by considering other potential variables than sociodemographic characteristics, work situation during the pandemic, and place of living. Although these characteristics are supposed to reflect specific life challenges and health, social, and economic concerns, other factors should be considered such as psychological well-being, longer or shorter life expectancy, perceived social support, trust in institutions, sense of belonging to the community, current intergenerational differences with respect to the sensitivity and interests expressed toward other social problems causing a catastrophic impact for the whole of humanity (e.g., climate change), and different exposure to social network communication to better understand how micro and macro social spheres influence the ways of interpreting the pandemic crisis.

Third, on the basis of SCPT and previous studies that have shown the essential role of SUs in grounding, motivating, and channeling social and individual behavior, we have suggested that SUs might favor or hinder an adaptive response to the crisis. However, the current study does not allow this relationship to be examined further. Further studies should longitudinally examine the variability of the SUs over time and their impact on psychological well-being and people responses to the crisis in the medium and long term (e.g., degree of compliance toward the health emergency measures established by the government and levels of engagement in solidarity actions).

## Conclusion

This article has explored the meaning of living in the time of COVID-19 through the collection of narratives from Italian adults and within the frame of the semiotic psychological theory of culture to enrich our understanding of the SUs active in the cultural milieu to interpret the current crisis.

The core of our proposal lies in the call to move beyond the idea that the pandemic can be taken for granted as being disruptive with a negative psychological impact on individuals and assume that those are the meanings through which people interpret their being-in-the-world to explain their reaction to the crisis, and that this reaction has to be understood in the light of their social–cultural milieu. What we need to do is to look more closely at the way individuals, their system of activity, and the sociocultural and political scenario interact with each other in constructing the impact of the pandemic on individuals and social life.

## Data Availability Statement

The raw data supporting the conclusions of this article will be made available by the authors, without undue reservation.

## Ethics Statement

The studies involving human participants were reviewed and approved by the project was approved by the Ethics Commission for Research in Psychology of the Department of History, Society and Human Studies of the University of Salento (protocol n. 53162 of 30 April 2020). The patients/participants provided their written informed consent to participate in this study.

## Author Contributions

CV and TM conceived the study and overall edited the manuscript. All the authors collected the data, organized the relevant literature, and interpreted the results. CV wrote the manuscript, with the contribution of TM. TM and AG conducted the data analysis. TM, AG, and AP reviewed the manuscript sections.

## Conflict of Interest

The authors declare that the research was conducted in the absence of any commercial or financial relationships that could be construed as a potential conflict of interest.

## Footnotes

1 http://www.aipass.org/node/26
